# The Hospital Saturday Fund's System of Distribution

**Published:** 1897-04-03

**Authors:** 


### The Institutional Workshop.

### THE HOSPITAL SATURDAY FUND'S SYSTEM OF DISTRIBUTION.

Commentiug upon an article on the above subject, which appeared in The Hospital on March 20th, Mr. Acland says :—

One "educated man" does not approve of making a grant to the Queen's Jubilee Hospital or of awarding it for economy "a sum nearly equal to the money granted for economy (by the way it is 27 per cent. less, but that is nothing) to the largest and on the whole, probably, the best administered hospital in the country." Because he objects he has called attention to it, and is trying to get the system altered. You, Mr. Editor, are an autocrat—all editors, I believe, are—but the mere chairman of a Hospital Saturday Fund is not, and can only call attention to defects, try and get them remedied, and apparently provide stones to be hurled at his organisation. The "educated man" to whom I refer—can it be we both mean the same person?—is R. B. D. Acland, Chairman of the Hospital Fund. Excuse my pointing out that I have never "advocated the general adoption by other funds of the plan of distribution devised by my committee," and this is perfectly obvious from the address which has given rise to your note. I am much too alive to the defects of our own system, which are I think clearly pointed out in my address, to advocate any such course, though, perhaps, you may be able to refer me to some occasion when I have done so. I have criticised other systems, and have said that I think a good system could be worked out by a consultation of all who have tried to work out a plan; indeed, I say in the next paragraph to that to which you have referred, that I do not think even now I have discovered a perfect system, and in the next but one express a hope that a conference between the council of the Prince of Wales's Fund, the Sunday, and Saturday Funds might result in a scheme being worked out which all might use. But any stick is good enough to beat a dog with.

*** The grant referred to, to the Queen's Jubilee Hospital, is so monstrous a thing in itself as to make one wonder that anyone with proper self-respect should allow himself to be identified with it in any way. If a man deliberately walks over a precipice and breaks his neck that is his own affair; but in public matters, where the distribution of public money is concerned, to remain a member of a body which improperly applies the funds entrusted to it by giving them to unworthy objects, is to participate in the responsibility of such conduct. That is why we ventured to express our astonishment at an educated man consenting to have his name identified with the system which permits a grant like that referred to. Our impression that Mr. Acland advocated the general adoption by other funds of the plan of distribution devised by his committee is based upon a speech made by him at the Mansion House on January 30th, 1896. The impression that speech conveyed was certainly that he desired to substitute the Hospital Saturday system, or a portion of it, by instructing the Distribution Committee of the Hospital Sunday Fund to alter the clause in the constitution accordingly. We are very glad indeed to hear that this impression was erroneous, and we take this opportunity of expressing our regret that we should have inadvertently attributed views to Mr. Acland which he does not hold. Mr. Acland has done such yeoman service by the patient self-effacement which has characterised his tenure of the chairmanship of the Hospital Saturday Fund, that he deserves the grateful recognition of all who are interested in the hospitals. His great patience has been exemplary, especially in regard to that crying abuse, street collections; but there is a point where patience may be interpreted as weakness, and, although we should not like to say Mr. Acland has ever reached that point, he must have been perilously near it when he permitted his name to be associated with the grant of £30 4s. 4d. for economy to that miscalled building, which goes by the name of the Queen's Jubilee Hospital. An act like this on the part of the Hospital Saturday Fund does more harm to the cause of hospitals in London than the whole of the sum they collect in any given year can do good, and the sooner the working men can be made to realise the truth of such facts the better. In the same way the street collections are a crying abuse, injurious to the hospitals, and more damaging to the reputation of the Hospital Saturday Fund than we care to express in words. We want the Hospital Saturday Fund to be a help to the hospitals and a credit to everybody; and Mr. Acland's whole career shows that this is his position, too. We hope, therefore, he may succeed in reforming the present system of distributing the funds of the Hospital Saturday Fund, and securing that the year of the Queen's commemoration shall not be disgraced by the holding of street collections in connection with the Hospital Saturday Fund throughout London. We fully appreciate the difficulties which beset Mr. Acland in his position of chairman, but the matter of the street collections is so serious that no step is too strong to take to secure their immediate abolition. For example, if Mr. Acland were to resign the chairmanship tomorrow because the street collections are to be held again this year in connection with the Hospital Saturday Fund, he would get such a backing in the public press and such a support from the public that it is probable the Hospital Saturday Fund would, if these collections were still held, disappear altogether from the organisations of the metropolis, and the contributions of the working classes would be given to hospitals in some other form.—Ed. *T.H.*]

